# Return of showjumping horses to sporting activity after colic surgery

**DOI:** 10.1111/evj.14407

**Published:** 2024-08-28

**Authors:** Gessica Giusto, Marco Gandini

**Affiliations:** ^1^ Dipartimento di Scienze Veterinarie Università di Torino Grugliasco Italy; ^2^ Equine Health Center Torino Italy

**Keywords:** colic, horse, performance, recovery, sport, surgery

## Abstract

**Background:**

The return to performance after colic surgery is crucial for competition horses. While studies have investigated racehorse performance following colic surgery by analysing racing participation and earnings, this approach does not apply to showjumping horses, leaving a gap in the literature regarding their objective performance evaluation.

**Objectives:**

To assess the short‐ and long‐term survival and return to performance in showjumping horses after colic surgery.

**Study design:**

Retrospective case series.

**Methods:**

Medical records of horses with acute colic requiring surgical treatment were analysed, and data for showjumping horses (Group 1) were retrieved. Telephone follow‐ups were conducted and national competition databases were used to collect pre‐ and postoperative showjumping competition entries for Group 1 and for randomly selected horses (Group 2) participating in the same competitions as a comparison group.

**Results:**

Of 253 horses undergoing colic surgery, 96 were recorded as showjumpers. The median long‐term survival was 2.73 (0.01–6.14) years. Among these horses, 59 were competing at the time of surgery, and of these, 46 (78%) returned to competition and 41 (89.1%) competed at the same or higher level, while 5 (10.9%) competed at a lower level. At a 2‐year follow‐up, 63.6% of the showjumping horses that underwent colic surgery were alive. No significant differences were observed in the level of competition and career length between horses, which underwent colic surgery, and the randomly selected comparison group.

**Main limitations:**

Small sample size and a single‐centre design.

**Conclusions:**

Showjumping horses can make a successful return to competition after colic surgery, with the majority performing at the same or higher level as before the procedure.

## 1 INTRODUCTION

The return of horses to sporting activity and performance after colic surgery is an important concern in the equestrian world. In the United States, 98.8% of horses are primarily used for hacking, competition, showing, or farm work.^1^ The potential for a return to use and performance is carefully considered in relation to the costs of surgery. When owners anticipate high veterinary expenses, predict worse athletic outcomes, or anticipate long recovery times after surgery, they are less likely to give consent for exploratory laparotomy in cases of colic.^2^ This decision not only impacts the owner's future plans for the horse's use but can also influence the decision to euthanise the animal.^2^, ^3^ Therefore, the expansion of knowledge of expected survival rates for different surgical procedures, the prevalence of complications, and the possibility of a successful return to sports is crucial.^1^, ^4^, ^5^

Several studies^1^, ^4^, ^5^, ^6^, ^7^, ^8^, ^9^, ^10^, ^11^, ^12^, ^13^, ^14^ have found that 75%–90% of horses resumed sports activity at their previous performance level after colic surgery. Various studies have analysed the racing participation, results and prize income in racing following colic surgery.^12^, ^13^, ^14^ However, this approach is only applicable to racehorses. Studies in mixed equine populations have focused on their ability to perform at or above preoperative levels or at the expected level of performance according to the owners' anticipation.^1^, ^4^, ^5^, ^6^, ^7^, ^8^, ^9^, ^10^, ^11^ Limited research has employed objective evaluation methods alongside owner opinions in diverse populations of sport horses.^4^, ^5^, ^6^, ^7^, ^8^, ^9^ These studies found a positive association between subjective and objective performance evaluations only in certain sports.^4^, ^9^ An objective assessment of the differences in pre‐ and postoperative performance levels in sport horses, other than racehorses, is needed. There is currently a lack of research examining the likelihood of showjumping horses returning to sporting activities after undergoing colic surgery. By investigating the probability of showjumping horses resuming their intended use and achieving their previous level of performance following colic surgery, this study aims to fill this gap. Our hypothesis was that showjumping horses discharged from the hospital after colic surgery would be able to return to their intended use and perform at a similar level as before the surgery, and their postoperative performances would be no different from horses of the same age and that competed at the same level. To explore this hypothesis, the primary objective of the current study was to compare the pre‐ and postoperative competition levels reached by showjumping horses that have undergone colic surgery and to compare the performance and length of competing activities between showjumping horses that have undergone colic surgery and that of a control population of showjumping horses of similar age and performance level, selected from national competition databases.

## 2 MATERIALS AND METHODS

The study involved two groups of horses. Group 1 (treated, showjumping horses) included horses presented with acute colic that required surgical treatment at the University of Turin Veterinary Teaching Hospital between January 2017 and March 2021 and defined as showjumping horses by owners at the time of admission. For this group, data on signalment, weight, intestinal pathology described at surgery, surgical procedures, reproductive activity at the time of surgery (for mares) and whether they were discharged or not from the hospital were retrieved. Where a horse underwent multiple surgeries, only the data regarding the last surgery were included for analysis. Short‐term survival was defined as the horse being alive at hospital discharge. Horses that were subjected to pre‐ and intraoperative euthanasia due to decisions made by either the owner or the surgeon were included,^15^ to mitigate any potential bias arising from owners' or surgeons' decisions. Thus, the short‐term survival rate was calculated by dividing the number of horses discharged from the hospital by the total number of horses that presented with acute colic and required surgical treatment.

Telephone interviews were conducted with the owners in April 2023. Questions asked were whether the horse was alive, in which activity it was employed, whether it was sold, or lost to follow‐up and the eventual reason for retirement from competitions or cause of death. The owners were asked whether the horse experienced post‐discharge colic, incisional infection or hernia, and whether these factors would have influenced the horse's return to competition. Long‐term survival was defined as the horse being alive at the time of the telephone interview performed at a minimum of 2 years after surgery and was expressed in years from the date of surgery. Survival rates at discharge, 1 and 2 years after surgery, were calculated for horses in Group 1.

A search was conducted on web‐based national competition databases Equiresults (www.equiresults.com; last accessed 31 March 2023) and Banca Dati Fise (https://www.fise.it/servizi/concorsi/concorsi-salto-ostacoli.html; last accessed 1 April 2023) for both pre‐ and postoperative entries in national and international showjumping competitions. Group 1 horses that had not competed at least once in the 12 months before surgery were considered as ‘not in activity at the time of surgery’. Horses were categorised as ‘retired after surgery’ if they had competed within the 12 months preceding the surgery, but no records were found in the databases after the date of emergency laparotomy. Horses were classified as ‘in activity after surgery’ if they had competed at least once within the 12 months before the surgery and also competed at least once after the date of emergency laparotomy. To define postoperative sporting activity, the timeframe used to search results in the national competition database was between the date of surgery and the date of analysis of data (1 April 2023) and provided a minimum of 2 years period.

Horses in Group 1 were categorised into four levels based on the highest competition they participated in during the 12 months before surgery (preoperative level) and the entire period after surgery (postoperative level). These levels were determined by the height of the obstacles: Level 1 represented pleasure competitions (up to 80 cm high), Level 2 denoted intermediate competitions (90–110 cm high), Level 3 indicated advanced competitions (115–130 cm high) and Level 4 referred to elite competitions (greater than 130 cm high). Two subgroups of horses were created based on their performance levels: Group 1H included horses that maintained the same or achieved higher levels of performance after surgery, while Group 1L comprised horses that performed at a lower level postoperatively compared to their preoperative level. This determination was made by comparing the difference between their highest postoperative and preoperative performance levels. Career length was determined as the duration between the surgery and the last recorded competition in the databases. Comparisons were conducted between the pre‐ and postoperative levels of performance for each horse in Group 1. In addition, a comparison of the postoperative levels of performance was made between horses in Group 1 that received resection and anastomosis during surgery or not.

A search was conducted for each horse in Group 1, which competed after surgery, using web‐based national competition databases Equiresults (www.equiresults.com; last accessed on 31 March 2023) and Banca Dati Fise (https://www.fise.it/servizi/concorsi/concorsi-salto-ostacoli.html; last accessed on 1 April 2023). The last competition participated in by these horses before their surgery date was analysed, and two horses of similar age (within a ±1 year age difference) were randomly selected from the starting list of the same competition using a web‐based program (https://www.random.org/lists/?mode=advanced). Subsequently, these 92 chosen horses were categorised into Group 2 (untreated, showjumping horses). To evaluate the performance level of the horses in Group 2, information on their performance before and after the surgery date, their age at the last recorded competition, and the length of their career, as previously defined, was retrieved. The data obtained for horses in Group 2 was compared with that of Group 1. To determine the length of the career, the date of surgery was set as the starting point. This approach was chosen because some horses in Group 2 might have eventually completed their careers during the period when the horses in Group 1 were hospitalised. Starting the timeline from the surgery date ensures a consistent and fair comparison for both groups. There was no direct contact with the owners of the Group 2 horses, and therefore, it is not known if they had undergone colic surgery or encountered any veterinary problems that might have impacted their career.

### 2.1 Data analysis

To determine the normal distribution of data, the Shapiro–Wilk test was used and since data were non‐normally distributed, non‐parametric tests were applied. Long‐term survival and career length were calculated, and survival curves were constructed using the Kaplan–Meier method. Horses lost to follow‐up were censored at the last time point they were observed. Log rank Mantel–Cox test was used to compare survival curves. The Mann–Whitney test was used to determine differences in age at retirement and length of postoperative career between Groups 1 and 2 at a definite time point (April 2023). All statistical analyses were conducted using a commercial software package (GraphPad Prism 9.0, GraphPad Inc., La Jolla, CA, USA), and statistical significance was set at *p* < 0.05.

## 3 RESULTS

### 3.1 Short‐ and long‐term follow‐up of horses which underwent colic surgery

**FIGURE 1:**
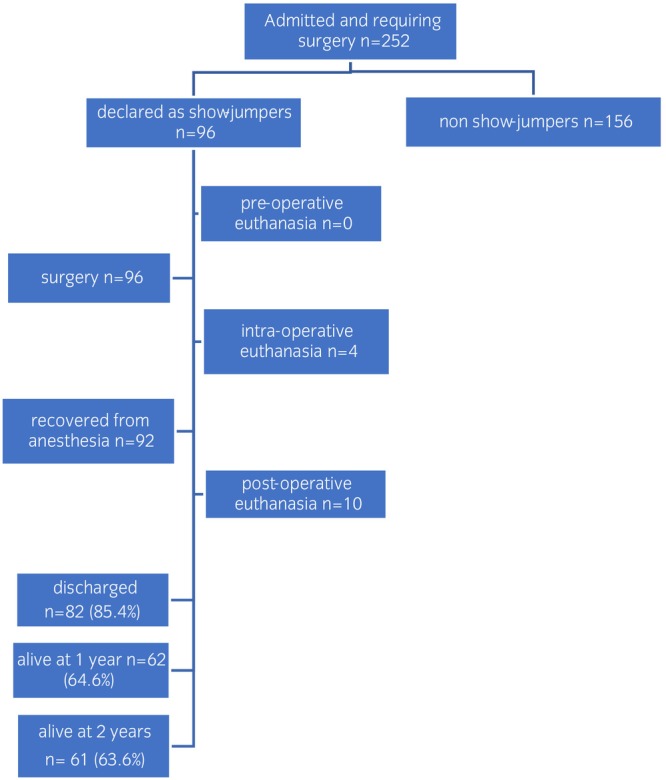
Flowchart describing the outcome of 253 horses admitted for acute colic requiring surgery at the University of Turin VTH.

From a total of 252 equine patients that presented with acute colic and required surgical treatment, 96 were showjumpers, according to their owners including 32 Italian saddle horses, 18 Belgian warmblood horses, 11 mixed‐breed horses, 10 Selle Francaise horses, 9 Hungarian saddle horses, 8 Koninklijk Warmbloed Paardenstamboek Nederland horses, 3 Zangersheide horses and 5 ponies and 43 were geldings, 39 were mares and 14 were stallions The median (range) age at the time of surgery was 13.95 (3.7–24.6) years, 30 (31.2%) were over 16 years of age. Five (5.2%) weighed <300 kg (ponies or miniatures) and none of the mares were used for breeding. Pathologies found at surgery or necropsy are detailed in Table 1 while outcomes of these horses are reported in Figure 1. Four horses were euthanised intraoperatively due to either viscera rupture or excessive length of non‐viable intestine. Twenty‐five (27.2%) horses underwent resection and anastomosis (nine jejunocecostomies, nine jejunojejunostomies, two hybrid jejunoileocecostomies,^16^ two total large colon resections, two wedge large colon resections and one ileocecolostomy), while nine underwent anastomosis only (incomplete ileocaecal bypass).

**TABLE 1:** Distribution of the different pathologies found at surgery or necropsy in showjumping horses admitted for colic surgery at the University of Turin VTH.

		Group 1 (*n*)
Large intestine	Right dorsal displacement of large colon	23
	Colon torsion	7
	Nephrosplenic entrapment	13
	Large colon impaction	11
	Small colon impaction	1
	Others (cecocolic intussusception, caecal intussusception, adhesions)	1
Small intestine	Entrapment in the epiploic foramen	11
	Ileal impaction	9
	Lipoma	0
	Inguinal hernia	6
	Gastrosplenic entrapment	5
	Others (adhesions, intussusception, DPJ, jejunal volvulus)	8

The short‐term survival rate was 85.4% (82 horses discharged of 96 admitted). Of the 34 showjumping horses that had anastomosis with or without resection, 26 were discharged from the hospital. Of these, five were not active at the time of surgery and five were retired after surgery. Median long‐term overall survival time was 2.73 (0.01–6.14) years.

Colic was identified as the cause of death in 10 of 21 fatalities, other causes were laminitis, pituitary pars intermedia dysfunction, and equine herpes virus myeloencephalitis in one horse each. Seven horses (11.3%) were reported to have postoperative colic after discharge, and the prevalence of surgical site infection was 13.1% (eight horses), while one horse (1.6%) developed an incisional hernia.

### 3.2 Pre‐ and postoperative performance of showjumping horses which underwent colic surgery

Of the 82 horses that were discharged from the hospital, 59 horses were competing at the time of surgery, of which 46 (78%) returned to competition within a median time of 8.8 months (range 3.5–33.3 months) (Figure S1). Thirteen horses that were active at the time of the surgery were retired thereafter. The age at surgery was a mean of 12.7 years (±4.16) years for those who went on to compete again after surgery and 14.9 (±3.57) years for those who were retired postoperatively.

**FIGURE 2:**
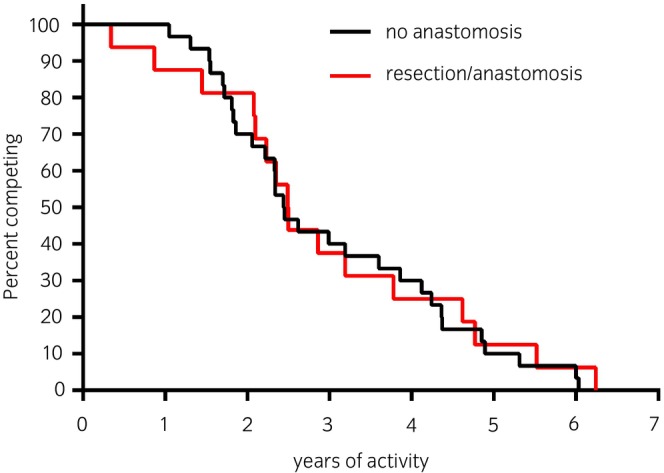
Kaplan–Meier plot of the length of the career of horses in Group 1 discharged from the hospital with or without anastomosis. Number of horses in Group 1 that returned to competition, *n* = 46; with anastomosis *n* = 16, without anastomosis *n* = 30, time 0 = date of discharge (log rank Mantel–Cox test, *p* = 0.7).

Of the horses that continued competing after surgery, the majority competed at the same or higher level (subgroup H), whereas only five (10.9%) competed at a lower level (subgroup L, Table 2) as compared to before surgery. Career length was not significantly different between subgroups H and L (Figure S2). Horses that competed at Level 1 preoperatively had a shorter postoperative career than those that competed at Levels 2 and 3 preoperatively (Figure S3). Horses competing at Level 1 postoperatively had a shorter career than horses that reached higher levels. All horses that competed at the elite level (i.e., Level 4) postoperatively had competed at a lower level before surgery. Of these, four of the five horses were ≤6 years old before surgery and all horses ≤6 years old that returned to competition competed at the same or higher level after surgery. The only horse that was competing at Level 4 preoperatively competed at a lower level after surgery. Career length was not different between horses with and without anastomosis (Figure 2). Hernia and surgical site infection were the cause for the delayed return to competing for one and two horses, respectively, according to the owners. Eight horses were lost to follow‐up. Of the 46 horses that returned to competition after surgery, 13 horses were no longer participating in competitions at the time of the telephone follow‐up. The owner‐reported reasons for not competing were recurrent colic in two horses, orthopaedic problems, and changes in ownership or activity related to the owner's choice in the remaining 11 horses.

**TABLE 2:** Number of showjumping horses in Group 1 per pre‐ and post‐competition level admitted for colic surgery at the University of Turin VTH.

Level	Height of jumps	No. of horse per level, preoperatively	Competed at lower level (Group L)	Competed at same or higher level (Group H)
1	<80 cm	4	0	4
2	81–110 cm	27	3	24
3	111–130 cm	14	1	13
4	>130 cm	1	1	0
Total number		46	5	41

### 3.3 Comparison between Group 1 (treated, showjumping horses) and Group 2 (randomly selected showjumping horses)

**FIGURE 3:**
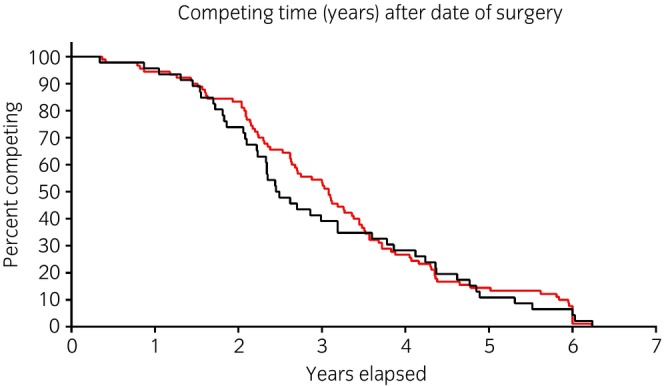
Kaplan–Meier‐plot of the length of career between horses in Groups 1 and 2. Number of horses 138 (46 Group 1, 92 Group 2), time 0 = date of surgery of horses in Group 1 (log rank Mantel–Cox test, *p* = 0.3). Black line: Group 1, red line: Group 2.

Ninety‐two horses were included in Group 2. The median (range) postoperative competition level was not significantly different between Group 1 (2, 1–4) and Group 2 (2, 1–4) and the age at retirement and career length did not differ significantly between Groups 1 and 2 (Table 3). Median career length was 2.5 (0.34–6.24) years for Group 1 and 3.08 (0.37–6.23) years for Group 2. The overall career length (Figure 3), and career length by competition level did not differ except for horses in Group 1 competing at Level 1 postoperatively, which had a shorter career than did horses in Group 2 competing at the same level (Figure S4, *p* = 0.006).

**TABLE 3:** Age at retirement and length of postoperative career between horses in Group 1 and Group 2 (years, Mann–Whitney test).

Postop level	Age at retirement (years)					Length of postoperative career (years)				
	*n*	Group 1	*n*	Group 2	*p* Value	*n*	Group 1	*n*	Group 2	*p* Value
1	6	13 (13–20)	9	16 (13–24)	0.22	6	1.6 (0.34–2.6)	9	3.4 (1.4–4.4)	**0.02**
2	18	16 (9.2–22)	44	16 (8.1–24)	0.98	18	2.4 (0.9–6.2)	44	2.7 (0.37–6.0)	0.28
3	17	17 (10–24)	36	16 (8.1–23)	0.358	17	3.2 (1.7–6.0)	36	3.5 (0.41–6.2)	0.88
4	5	12 (9.9–14)	3	19 (11–20)	0.25	5	4.6 (3.6–6)	3	2.3 (1.9–4.8)	0.39
Total	46	15.5 (9.2–24)	92	15.9 (8.9–24.1)	0.56	46	2.45 (0.34–6.24)	92	3.08 (0.37–6.23)	0.27

## 4 DISCUSSION

No previous study has specifically and objectively investigated the return to activity of showjumping horses with respect to survival, career length and pre‐ and postoperative competing levels. In our study, the definition of competition activity and the level of competition before and after surgery was not reliant on a questionnaire. This approach was adopted to minimise the potential influence of subjectivity in the owners' responses, which has been previously reported in other studies.^12^ National competitions database searches to obtain competition entries and competition level offered an objective tool to define the level of performance and length of career, as well as the time between surgery and return to competition.

Survival in the short term was comparable to other studies. The observed survival rate and the median survival time at the time of follow‐up compare favourably with what has been reported in the literature.^3^, ^4^, ^5^, ^6^, ^7^, ^10^, ^15^, ^17^ This finding is noteworthy, considering that, in accordance with the recommendations of Freeman,^15^ the short‐term survival rate was calculated based on the number of horses that were initially deemed suitable for colic surgery upon admission. This was done to reduce bias due to pre‐ and intraoperative euthanasia, either due to case selection or financial constraints.

Of the horses that were active at the time of surgery and subsequently discharged from the hospital, the majority successfully returned to competition. When considering the percentage of horses that performed at the same level or even improved after colic surgery, these results compare favourably with findings from other studies.^1^, ^4^, ^5^, ^6^, ^7^, ^8^, ^9^, ^10^, ^11^, ^12^, ^13^, ^14^ The median return to competition after surgery was 8.8 months, which was not different from that reported in other studies.^4^

The length of a career remains a difficult trait to define in showjumping, as it depends on the age of the horse at debut and is frequently affected by the choices of the owner due to the ‘amateur’ status of the sport. Due to the limitations of national databases, which only provided results dating back to 2015, it was not feasible to accurately determine the complete length of the horses' careers, as conducted in other studies.^18^ However, we focused on assessing the duration of the horses' careers, specifically after undergoing colic surgery. In the context of this study, this aspect held greater significance than considering the entirety of their careers, as the starting age of showjumping horses can vary.^19^, ^20^ The median length of career was similar for horses in Group 1 and Group 2 and in excess of 2 years. Furthermore, the length of the postoperative career was not significantly different either between Group 1H and Group 1L, further demonstrating no effect of colic surgery on the competition performance of treated horses. For medium‐to‐high levels of competition, career length did not differ significantly between Groups 1 and 3; the career length of horses in Group 1 competing at Level 1 before and after surgery was significantly shorter than that of horses in Group 3 competing at the same level. The explanation could be that these horses are often ridden by young riders who change horses over time, by amateur riders who eventually change sports, or by the fact that owners consider surgery an extremely stressful event to continue the sporting activity of the horse (as confirmed by telephone follow‐up).

Our study demonstrates that colic surgery did not have an impact on the career progression of horses. We observed instances where horses, despite competing at a lower level or being horses of a younger age before surgery, were able to participate in elite competitions after undergoing the surgical procedure. Furthermore, causes for retirement after surgery or for no longer being active at the time of follow‐up were mostly reported by owners to be related to orthopaedic problems or owner preferences.

The complexity of surgery did not affect the careers of showjumping horses. Approximately one‐third of horses that returned to competing after colic surgery had an anastomosis performed during surgery. Among this group, the majority (60%) successfully returned to competition, including two horses that underwent a complete resection of the large colon. Career length was comparable to that of horses that did not receive an anastomosis. Telephone follow‐up revealed that owner‐reported incidence of postoperative complications was lower in our study than that reported in other studies,^4^ and the number of horses presenting with colic in the 2 years following the operation was lower than those reported previously.^1^, ^5^, ^9^, ^21^ This may be due to the selection of cases, different intra‐ and postoperative management between different referral centres,^22^ or other factors such as geographic differences. Nevertheless, as previously reported by Davis et al.,^1^ hernia and surgical site infection were found to be reasons for delays in returning to competition in three horses, while colic was identified as a cause for retirement in two horses.

This study has several limitations, including the small population size, the relatively limited number of elite horses and data obtained from one referral centre with some missing data that could impact the interpretation of the results. Since a very small number of horses underwent a second surgery, the impact of relaparotomy on performance has not been evaluated. It is worth noting that variations in the length of the postoperative period (among horses from 2017 to 2021) could potentially impact the overall length of horses' careers. Stratifying the career length based on the year of surgery would have resulted in small numbers, making it impractical for meaningful comparisons. A control for the difference in the length of the postoperative period between horses from 2017 and horses from 2021 when defining and comparing career lengths was not made. Whilst this may be regarded as a limitation, it is possible that the recorded length of career may be underestimated due to this unaccounted variation. We did not contact the owners of horses in Group 2 to ascertain whether the horses had undergone colic surgery or had other veterinary problems, and we cannot rule out the possibility that some of these horses had undergone colic surgery.

In conclusion, our results demonstrated that it is possible for showjumping horses to return to competition after colic surgery, with most horses competing at the same or higher level than before surgery. Colic surgery does not hinder the progress of young horses or limit the potential for horses to compete at high levels. In this study, resection and anastomosis did not appear to impair the ability of jumping horses to compete at the same performance level after surgery. Since a return to sporting activity may be a criterion for deciding whether to elect to perform colic surgery, the findings of this study may be useful to owners, referring veterinarians and surgeons to improve survival.

## FUNDING INFORMATION

Not applicable.

## CONFLICT OF INTEREST STATEMENT

The authors declare no conflict of interest.

## AUTHOR CONTRIBUTIONS

**Gessica Giusto:** Conceptualization; data curation; methodology; project administration; writing – original draft; writing – review and editing. **Marco Gandini:** Conceptualization; data curation; methodology; project administration; supervision; validation; writing – original draft; writing – review and editing.

## DATA INTEGRITY STATEMENT

Gessica Giusto had full access to all the data in the study and takes responsibility for the integrity of the data and the accuracy of the data analysis.

## ETHICAL ANIMAL RESEARCH

Research ethics committee oversight not required by this journal: retrospective study of clinical records.

## INFORMED CONSENT

Informed consent was obtained from the owners of all clinical cases at hospital admission. Data from publicly available sources was used without owner consent.

### PEER REVIEW

The peer review history for this article is available at https://www.webofscience.com/api/gateway/wos/peer-review/10.1111/evj.14407.

## Supporting information


**Figure S1.** Kaplan–Meier plot of the length of career of Groups 1H and 1L following colic surgery. Number of horses 46 (same or higher postoperative level, Group 1H, *n* = 41, lower postoperative level, Group 1L, *n* = 5), time 0 = date of discharge (log rank Mantel–Cox test, *p* = 0.7).

**Figure S2.** Kaplan–Meier‐plot of length of career for horses in Group 1 divided by preoperative competition levels. Number of horses 46 (preoperative level 1 *n* = 4, preoperative level 2 *n* = 27, preoperative level 3 *n* = 14, preoperative level 4 *n* = 1, the horse in this level was not included in the survival analysis because being a single case), time 0 = date of discharge (log rank Mantel–Cox test, *p* = 0.2).

**Figure S3.** Kaplan–Meier plot of career length for horses in Group 1 divided by postoperative competition levels. Number of horses 46 (postoperative level 1 *n* = 6, postoperative level 2 *n* = 18, postoperative level 3 *n* = 17, postoperative level 4 *n* = 5), time 0 = date of surgery time 0 = date of discharge (log rank Mantel–Cox test, *p* = 0.006).

**Figure S4.** Kaplan–Meier plot of length of career of horses in Groups 1 (continuous line) and 2 (interrupted line). Number of horses 138 (46 Group 1, 92 Group 2). (A) postoperative level 1: Group 1 *n* = 6, Group 2 *n* = 9 (log rank Mantel–Cox test, *p* = 0.006); (B) postoperative level 2: Group 1 *n* = 18, Group 2 *n* = 44 (log rank Mantel–Cox test, *p* = 0.4); (C) postoperative level 3: Group 1 *n* = 17, Group 2 *n* = 36 (log rank Mantel–Cox test, *p* = 0.8); (D) postoperative level 4: Group 1 *n* = 5, Group 2 *n* = 3 (log rank Mantel–Cox test, *p* = 0.4), time 0 = date of surgery of horses in Group 1.

## Data Availability

The data that support the findings of this study are available from the corresponding author upon reasonable request: Open sharing exemption granted by editor for this descriptive retrospective clinical report.
