# Supplementary material for: Return of showjumping horses to sporting activity after colic surgery
Source: Equine Vet J. 2024 Aug 28;57(3):629–35. doi: 10.1111/evj.14407 (PMC11982423; doi:10.1111/evj.14407)

Equine Veterinary Journal BEVA

Figure S1: Kaplan-Meier-plot of the length of career of groups 1H and 1L following colic surgery. Number of horses 46 (same or higher postoperative level, Group 1H, n=41, lower postoperative level, Group 1L, n=5), time 0 = date of discharge (Log-rank Mantel-Cox-test, p=0.7).

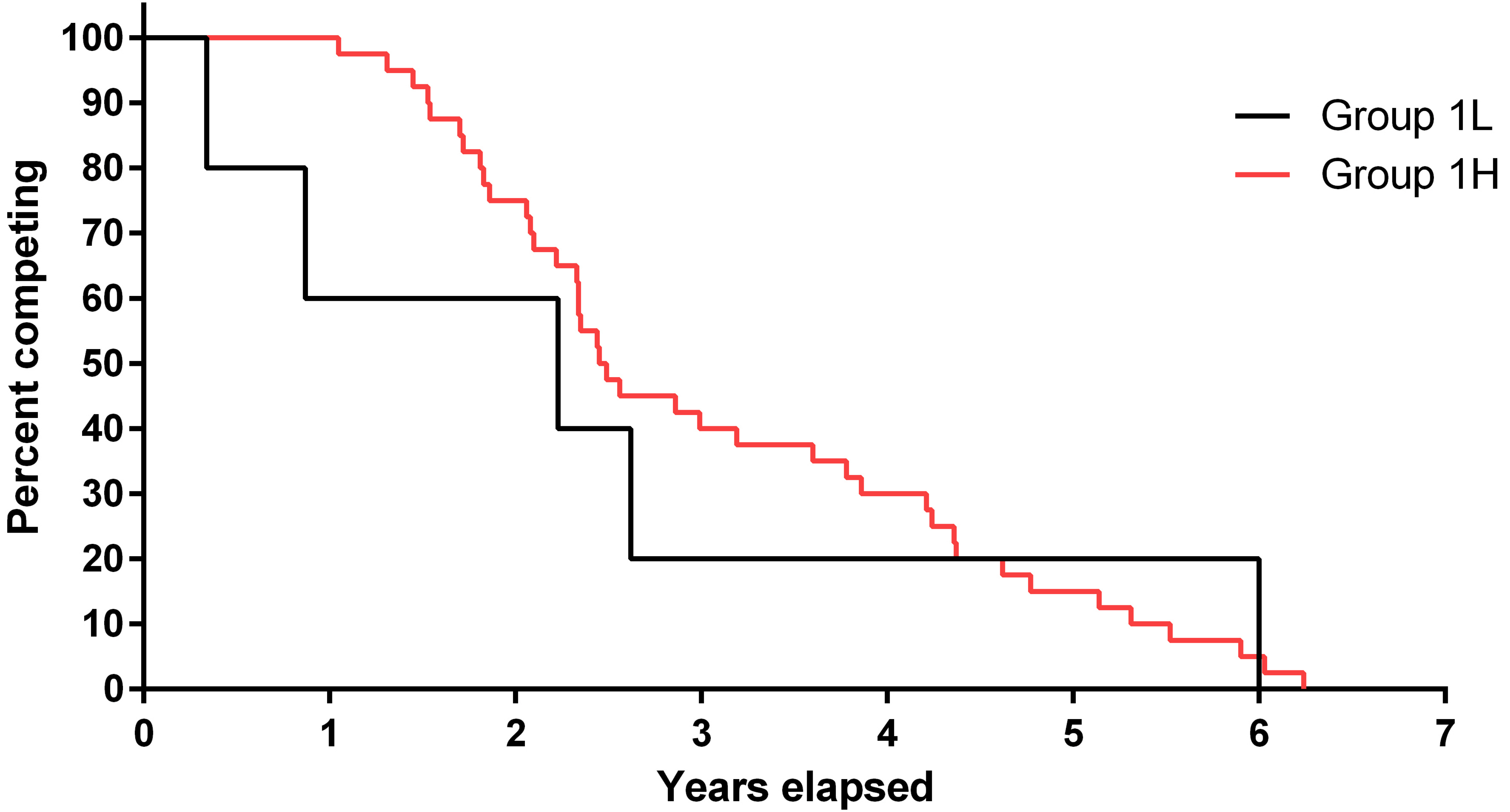

Supplement: Supplementary file 1 — Figure S1. Kaplan–Meier plot of the length of career of Groups 1H and 1L following colic surgery. Number of horses 46 (same or higher postoperative level, Group 1H, n = 41, lower postoperative level, Group 1L, n = 5), time 0 = date of discharge (log rank Mantel–Cox test, p = 0.7). [file EVJ-57-629-s003.pdf]
