# Supplementary material for: Return of showjumping horses to sporting activity after colic surgery
Source: Equine Vet J. 2024 Aug 28;57(3):629–35. doi: 10.1111/evj.14407 (PMC11982423; doi:10.1111/evj.14407)

Equine Veterinary Journal

BEVA

Figure S2: Kaplan-Meier-plot of length of career for horses in Group 1 divided by preoperative competition levels. Number of horses 46 (preoperative level 1 n=4, preoperative level 2 n=27, preoperative level 3 n=14, preoperative level 4 n=1, the horse in this level was not included in the survival analysis because being a single case), time 0 = date of discharge (Log-rank Mantel-Cox-test, p=0.2).

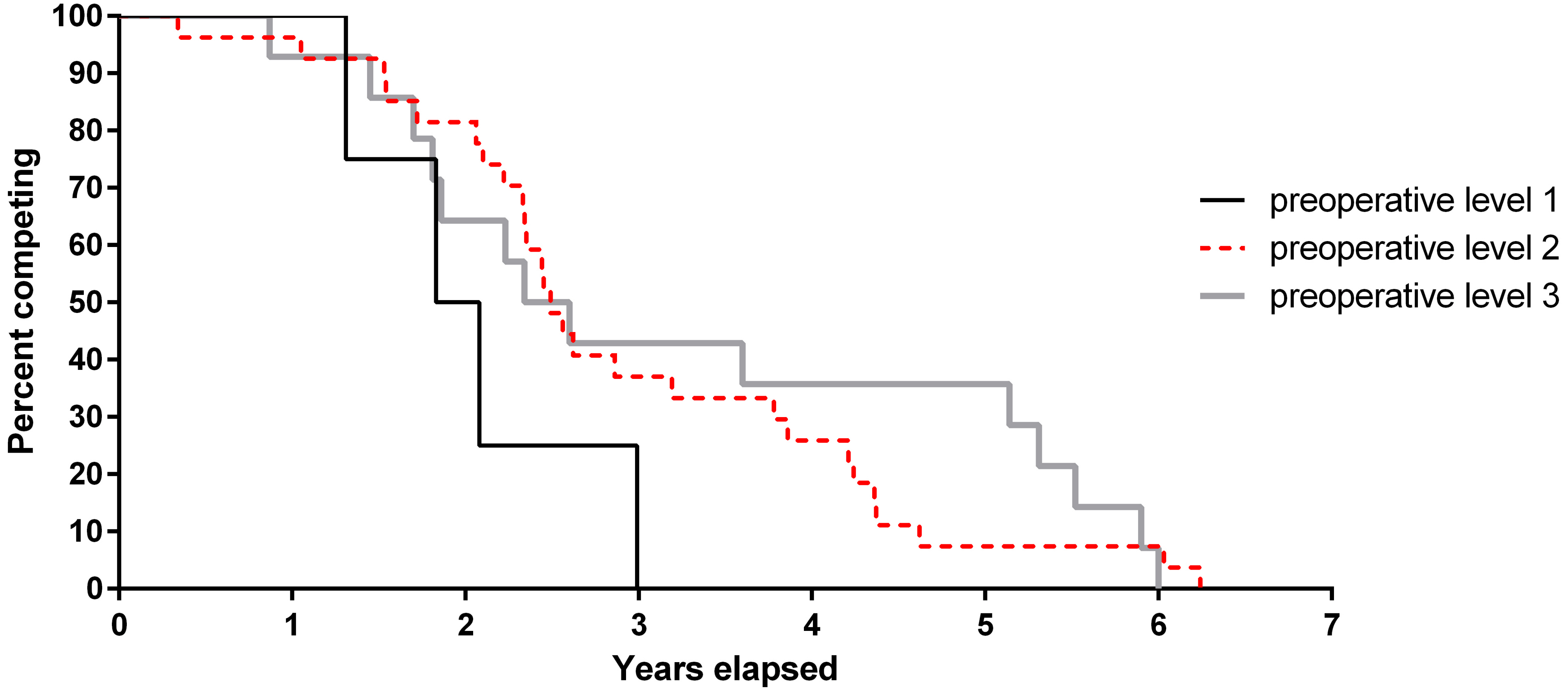

Supplement: Supplementary file 2 — Figure S2. Kaplan–Meier‐plot of length of career for horses in Group 1 divided by preoperative competition levels. Number of horses 46 (preoperative level 1 n = 4, preoperative level 2 n = 27, preoperative level 3 n = 14, preoperative level 4 n = 1, the horse in this level was not included in the survival analysis because being a single case), time 0 = date of discharge (log rank Mantel–Cox test, p = 0.2). [file EVJ-57-629-s004.pdf]
