# Supplementary material for: Return of showjumping horses to sporting activity after colic surgery
Source: Equine Vet J. 2024 Aug 28;57(3):629–35. doi: 10.1111/evj.14407 (PMC11982423; doi:10.1111/evj.14407)
Supplement: Supplementary file 3 — Figure S3. Kaplan–Meier plot of career length for horses in Group 1 divided by postoperative competition levels. Number of horses 46 (postoperative level 1 n = 6, postoperative level 2 n = 18, postoperative level 3 n = 17, postoperative level 4 n = 5), time 0 = date of surgery time 0 = date of discharge (log rank Mantel–Cox test, p = 0.006). [file EVJ-57-629-s002.pdf]

Equine Veterinary Journal BEVA

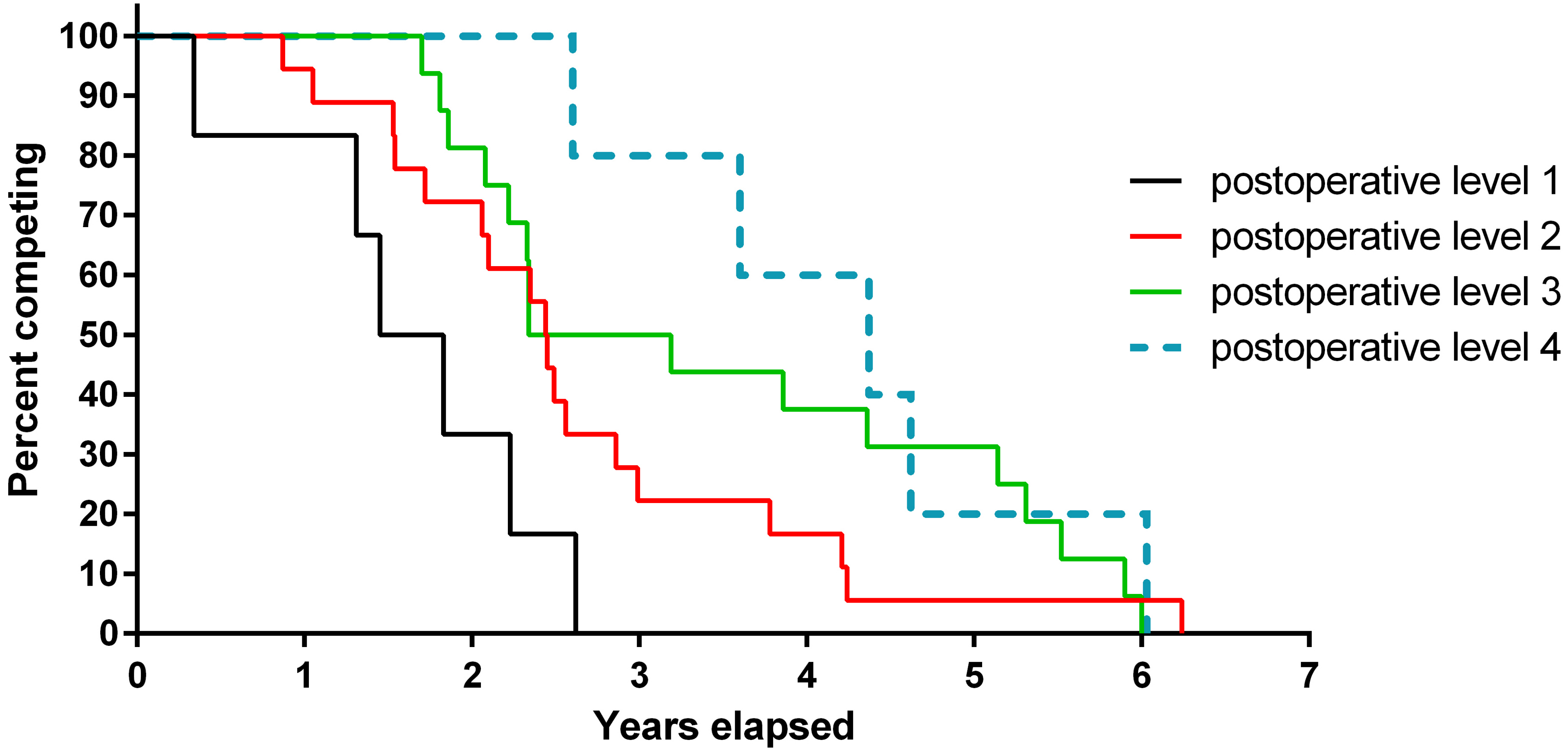

Figure S3: Kaplan-Meier-plot of career length for horses in Group 1 divided by postoperative competition levels. Number of horses 46, (postoperative level 1 n=6, postoperative level 2 n=18, postoperative level 3 n=17, postoperative level 4 n=5), time 0 = date of surgery time 0 = date of discharge (Log-rank Mantel-Cox-test, p=0.006).
