# Supplementary material for: Return of showjumping horses to sporting activity after colic surgery
Source: Equine Vet J. 2024 Aug 28;57(3):629–35. doi: 10.1111/evj.14407 (PMC11982423; doi:10.1111/evj.14407)
Supplement: Supplementary file 4 — Figure S4. Kaplan–Meier plot of length of career of horses in Groups 1 (continuous line) and 2 (interrupted line). Number of horses 138 (46 Group 1, 92 Group 2). (A) postoperative level 1: Group 1 n = 6, Group 2 n = 9 (log rank Mantel–Cox test, p = 0.006); (B) postoperative level 2: Group 1 n = 18, Group 2 n = 44 (log rank Mantel–Cox test, p = 0.4); (C) postoperative level 3: Group 1 n = 17, Group 2 n = 36 (log rank Mantel–Cox test, p = 0.8); (D) postoperative level 4: Group 1 n = 5, Group 2 n = 3 (log rank Mantel–Cox test, p = 0.4), time 0 = date of surgery of horses in Group 1. [file EVJ-57-629-s001.pdf]

Equine Veterinary Journal

BEVA

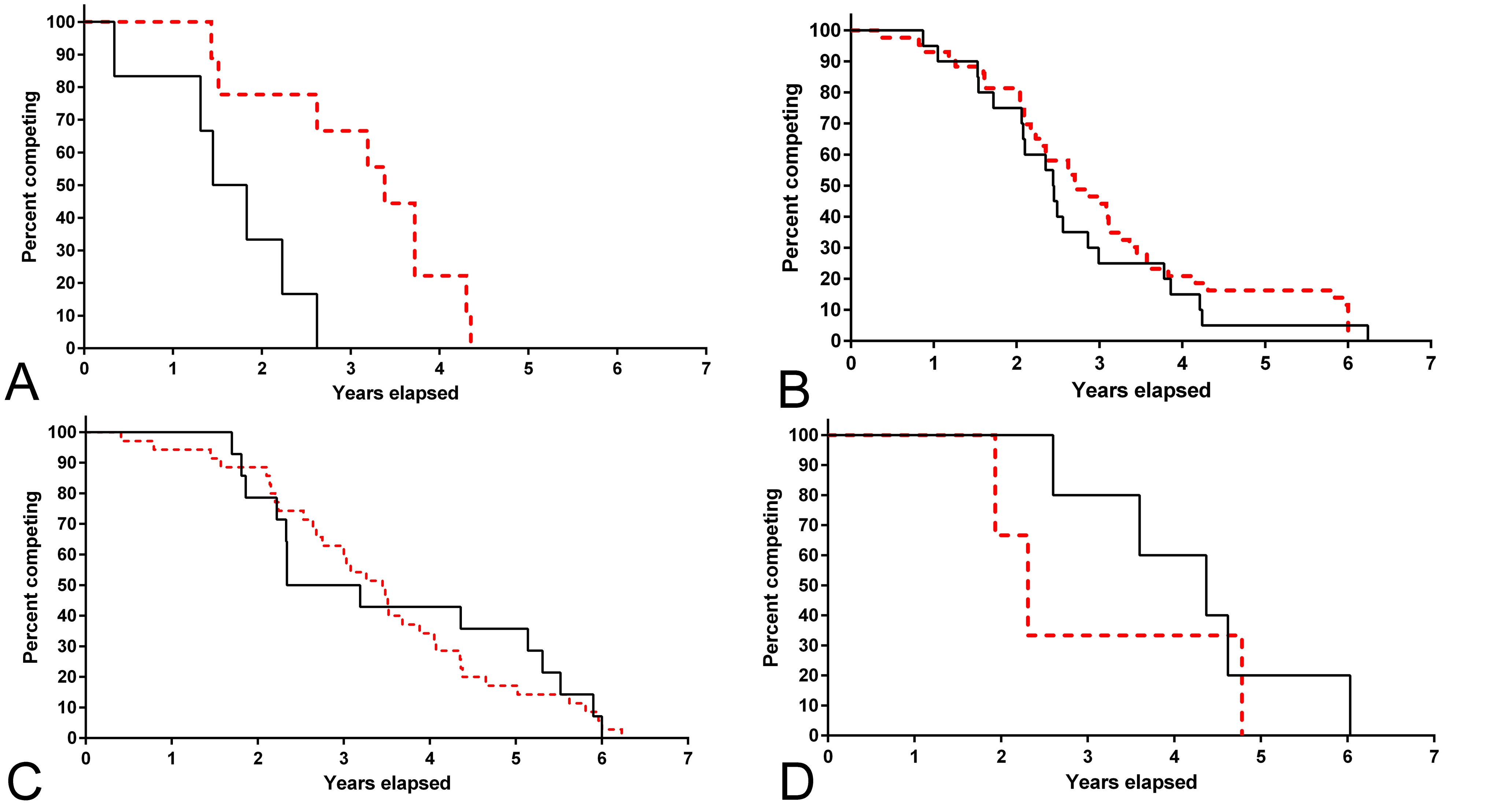

Figure S4: Kaplan-Meier-plot of length of career of horses in Group 1 (continuous line) and 2 (interrupted line). Number of horses 138 (46 Group 1, 92 Group 2). A) postoperative level 1: Group 1 n=6, Group 2 n=9 (Log-rank Mantel-Cox-test, p=0.006); B) postoperative level 2: Group 1 n=18, Group 2 n=44 (Log-rank Mantel-Cox-test, p=0.4); C) postoperative level 3: Group 1 n=17, Group 2 n=36 (Log-rank Mantel-Cox-test, p=0.8); D) postoperative level 4: Group 1 n=5, Group 2 n=3 (Log-rank Mantel-Cox-test, p=0.4), time 0 = date of surgery of horses in Group 1.
